# Face mask wearing image dataset: A comprehensive benchmark for image-based face mask detection models

**DOI:** 10.1016/j.dib.2023.109755

**Published:** 2023-11-04

**Authors:** Yogesh Suryawanshi, Vishal Meshram, Vidula Meshram, Kailas Patil, Prawit Chumchu

**Affiliations:** a Vishwakarma University, India; b Vishwakarma Institute of Information Technology, India; c Kasetsart University, Thailand

**Keywords:** Classification, Deep learning, Facemask images, Image analysis, Machine learning, Public health

## Abstract

The Face Mask Wearing Image Dataset is a comprehensive collection of images aimed at facilitating research in the domain of face mask detection and classification. This dataset consists of 24,916 images, carefully categorized into two main folders: “Correct” and “Incorrect” representing instances of face masks being worn properly and improperly, respectively. Each folder is further divided into four subfolders, each denoting a specific type of face mask - Bandana, Cotton, N95, and Surgical. In the “Correct” folder, images depict individuals correctly wearing their respective face masks, while the “Incorrect” folder contains images of improper face mask usage. To capture variations in face mask application across different demographics, such as age and gender, each subfolder also includes three additional subfolders - Child, Male, and Female. The dataset's diverse content encompasses different face mask types, covering bandana-style, cloth, N95 respirators, and surgical masks, across various age groups and genders. This design ensures a comprehensive representation of real-world scenarios, enabling the evaluation of machine learning algorithms for face mask detection and classification. Researchers can leverage this dataset to develop and assess models that can accurately identify and distinguish between correct and incorrect face mask usage. By contributing to the advancement of face mask detection technologies, this dataset further supports public health initiatives and encourages proper mask-wearing behavior to mitigate the spread of infectious diseases, particularly during times of heightened health concerns such as the COVID-19 pandemic.

Specifications Table

**Table:** 

Subject	Applied Machine Learning, Global Pandemic
Specific subject area	Face mask classification
Data format	Raw
Type of data	Images
Data collection	The Face Mask Wearing Image Dataset is a comprehensive collection of images, meticulously categorized into “Correct” and “Incorrect” folders to represent proper and improper face mask usage, respectively. It includes four subfolders for each mask type (Bandana, Cotton, N95, and Surgical), showcasing individuals wearing masks correctly or incorrectly. Moreover, subfolders based on gender (Child, Male, Female) capture diverse scenarios. The dataset comprises 24,916 high-definition images, captured at various angles and in different environments using mobile phone cameras with a size of 1280×768 pixels. These images provide valuable resources for training and evaluating face mask detection and classification algorithms.
Data source location	1. Vishwakarma University, Kondhwa Budruk, Pune – 411048. Maharashtra, India. 2. Latitude and longitude: 18.4603°N, 73.8836°E 3. Hubtown Countrywoods, Tilekar Nagar, Kondhwa Budruk, Pune – 411048. Maharashtra, India. Latitude and longitude: 18°26′35.9″N, 73°53′02.9″E
Data accessibility	Repository name: Face Mask Wearing Image Dataset: Correct vs. Incorrect Usage Data identification number: 10.17632/8pn3hg99t4.2 Direct URL to Data: https://data.mendeley.com/datasets/8pn3hg99t4/2

## 1 Value of the Data

• Facilitating Face Mask Detection Research: It enables the development and evaluation of accurate face mask detection and classification algorithms.
• Diverse Scenarios and Demographics: The dataset captures varied face mask types, age groups, and genders, making it a comprehensive benchmark for real-world scenarios.
• Supporting Public Health Initiatives: With the ongoing importance of face masks in disease control, the dataset aids in monitoring compliance and effective public health strategies.
• Enhancing Model Generalization: Its diverse images promote the development of robust face mask detection models that can handle various lighting conditions and backgrounds.
• Benchmarking and Advancements: The standardized benchmark facilitates healthy competition among researchers, encouraging advancements in face mask detection technology

## 2 Data Description

The objective of the “FaceMask detection” project is to create a high-quality image dataset representing various face mask usage scenarios under diverse lighting conditions and backgrounds. This dataset aims to train machine learning models for accurate face mask detection and classification, enabling real-time automation of operations like sorting and quality control in the context of face masks. By fostering responsible mask usage and supporting public health initiatives, the project seeks to contribute to improved mask-wearing behavior and effective disease control measures. This dataset can be used in current or future global pandemic situations.

The FaceMask detection dataset is a meticulously curated collection of high-quality images focusing on various aspects of face mask usage. It encompasses individuals wearing different types of face masks, namely Bandana, Cotton, N95, and Surgical, under diverse scenarios. Each type of face mask is further classified based on proper and improper usage, resulting in two main categories: “Correct” and “Incorrect” Within the “Correct” category, images depict individuals properly wearing their respective face masks, showcasing the correct way of donning each mask type. Conversely, the “Incorrect” category comprises images of improper face mask usage, capturing instances of mask misalignment or incomplete coverage. To ensure comprehensive coverage, the dataset includes diverse demographic representations, featuring individuals of different ages and genders in separate subfolders such as Child, Male, and Female for each face mask type. The images were meticulously captured in varying lighting conditions, including artificial light and natural light, to enhance model robustness under different illumination settings.

**Fig 1:**
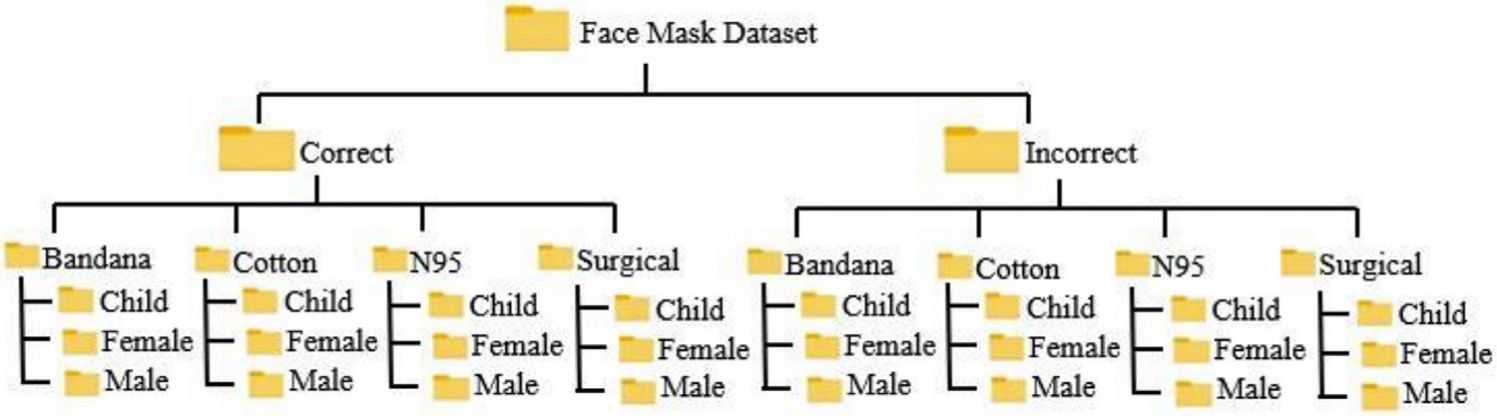
Directory structure of the FaceMask dataset.

**Fig 2:**
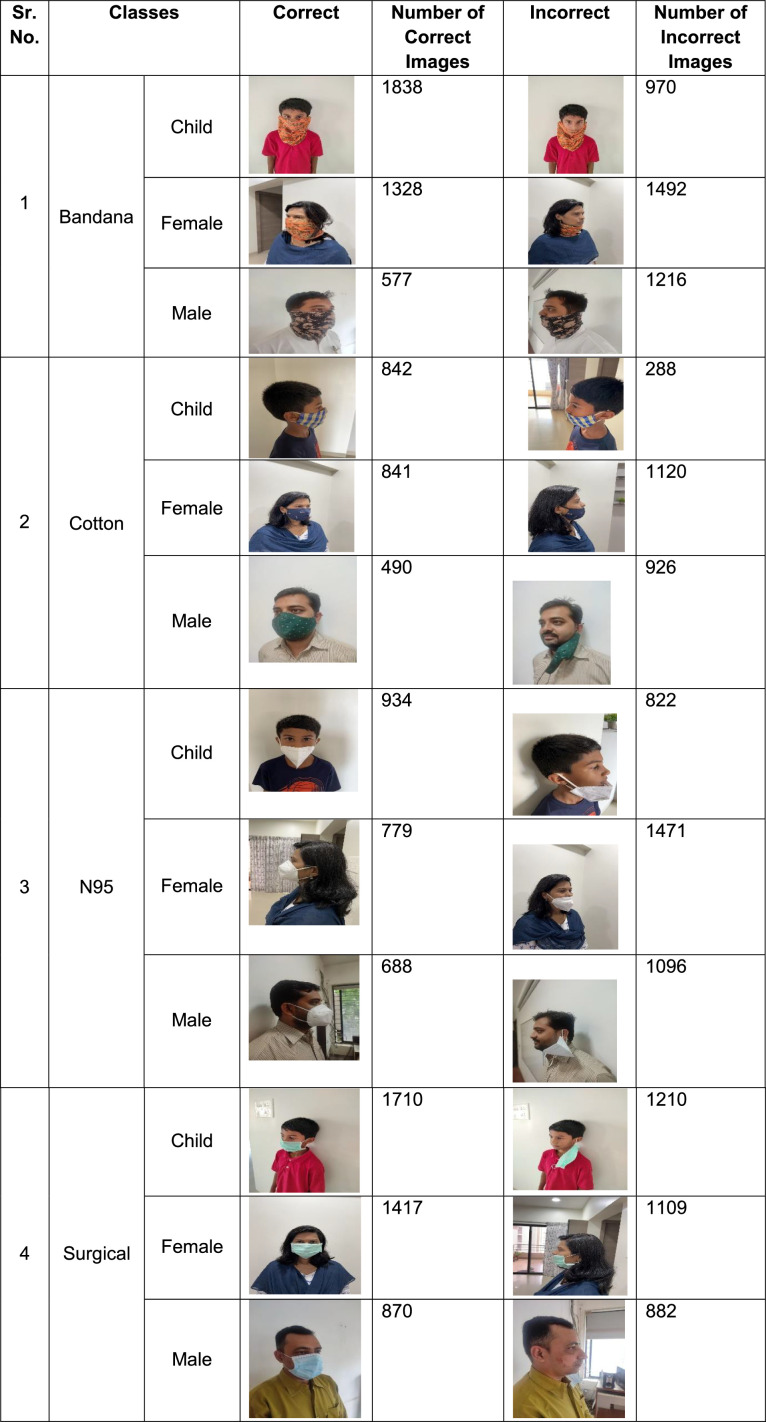
Sample images and their numbers from FaceMask dataset.

Fig. 1 showcases the hierarchical directory structure of the FaceMask detection dataset, ensuring seamless organization and ease of accessibility for researchers and developers. Selected sample images and the number of images per folder from the dataset are presented in Fig. 2, providing a representative glimpse of the dataset's content, quality and quantity.

## 3 Experimental Design, Materials and Methods

Image datasets have a pivotal role in diverse fields, encompassing computer vision and machine learning, as well as medical research and social sciences [[1], [2], [3], [4], [5], [6]]. The Face Mask Correct and Incorrect Wearing Image Dataset was created through a carefully designed experimental approach. We selected various face mask types, including Bandana, Cotton, N95, and Surgical masks, to encompass a wide range of mask variations commonly used in different settings. To ensure comprehensive coverage, participants from different demographics, including Male, Female, and Child, were involved in the study. These participants were photographed wearing the selected face masks both correctly and incorrectly, resulting in a dataset capturing real-life scenarios of proper and improper face mask usage. Following the methodology outlined by Lee et al. (2020) and WHO, participants engaged in hand hygiene practices before choosing properly fitted masks for the accurate depiction of correct face mask wearing. They then positioned the masks with the mask's colored side outward and, ensuring the metal strip rested atop the nose. The participants did the proper adjustment of elastic bands, along with firm pressing of the metal strip to match facial contours, enabled secure fitment of the mask. Thoroughly ensuring the absence of gaps between the face and mask, comprehensive coverage of the nose, mouth, and chin was underscored in the context of correct face mask wearing [12], [13], [14], [15]. The correct/proper way of wearing a face mask involves ensuring that the mask fully covers the nostrils conversely, an incorrect wearing of a face mask is demonstrated when the mask exposes or leaves the nostrils open in various ways.

**Fig 3:**
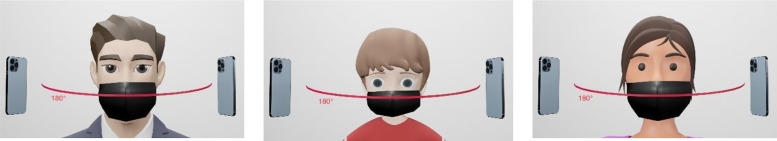
Illustrated representation of Face Mask Image methodology.

Both correct and incorrect face mask wearing photographs were captured using mobile phones, namely MI10TPro and Realme10 Pro 5G. For the creation of the face mask correct and incorrect wearing images, we undertook the wearing of various facemask types, including Bandana, Cotton, N95, and Surgical masks, by participants of different genders and age groups, namely Male, Female, and Child. Both correct and incorrect ways of wearing the facemasks were demonstrated, and photographs were captured from the anterior side of the face at a 180° angle (Fig. 3).

The face mask images were expertly captured using a high-quality 12 MP camera, boasting an impressive F stop of f/1.8 to 1.9, and an exposure time of 1/33 s to 1/50 s. The chosen focal length of 5 mm ensured sharpness and clarity in the images, allowing for detailed examination of the various face mask types and their correct and incorrect wearing positions. All images were subsequently converted to uniform dimensions of 1280 × 768 pixels and 96 dpi using IrfanView software, ensuring consistency and simplifying data processing. The images were then sorted and stored in respective folders based on face mask types and correct or incorrect face mask positions. The dataset was structured into two main categories: “Correct” consisting of images depicting individuals wearing their respective face masks correctly as outlined by Lee et al. (2020) and WHO [12], [13], [14], [15], and “Incorrect” comprising images showing improper mask usage, capturing instances of misalignment or incomplete coverage.

To ensure the dataset's diversity, additional subfolders were created for each face mask type, representing Child, Male, and Female participants. This demographic representation aimed to reflect different age groups and genders, contributing to a comprehensive and inclusive dataset. Moreover, to account for varying lighting conditions, the images were captured in artificial and natural light settings, enhancing the model's robustness and performance under different illumination scenarios. The dataset, comprising a total of 24,916 high-definition images, is now made available on Mendeley data, facilitating access for further research and analysis in the domain of face mask detection and classification [7].

The Face Mask Correct and Incorrect Wearing Image Dataset holds immense utility in the realms of computer vision, public health, and research. By training machine learning algorithms with this diverse collection of images depicting different face mask types and usage scenarios, it can facilitate the development of accurate models for face mask detection and classification. These models can be deployed in various environments to monitor compliance, encourage proper mask-wearing behavior, and contribute to public health initiatives during health crises like the COVID-19 pandemic. Moreover, the dataset can aid researchers and policymakers in studying mask efficacy, identifying common usage errors, and developing evidence-based policies to promote safe mask practices. With its potential for model improvement, awareness campaigns, and informed decision-making, this dataset serves as a valuable tool in fostering a culture of responsible and effective face mask usage [8], [9], [10], [11].

## Ethics Statement

The authors of this dataset, namely Yogesh Suryawanshi, Kailas Patil, and Vidula Meshram, are depicted in the dataset images. The authors and child parents have willingly given written informed consent for their inclusion in the study and have agreed to the public sharing of their image data. The authors declare no conflict of interest. This research did not involve animal or human studies and did not inflict harm on any living organism.

## CRediT authorship contribution statement

**Yogesh Suryawanshi:** Conceptualization, Data curation, Methodology. **Vishal Meshram:** Data curation, Writing – original draft. **Vidula Meshram:** Validation, Writing – review & editing. **Kailas Patil:** Supervision, Validation, Writing – review & editing. **Prawit Chumchu:** Validation, Writing – review & editing.

## Data Availability

Face Mask Wearing Image Dataset: Correct vs. Incorrect Usage (Original data) (Mendeley Data).

## References

[1] Suryawanshi Y., Patil K., Chumchu P. (2022). VegNet: dataset of vegetable quality images for machine learning applications. Data Br..

[2] Suryawanshi Y., Gunjal N., Kanorewala B., Patil K. (2023). Yoga dataset: a resource for computer vision-based analysis of Yoga asanas. Data Br..

[3] Meshram V., Patil K., Chumchu P. (2022). Dataset of Indian and Thai banknotes with annotations. Data Br..

[4] Meshram V., Patil K. (2022). FruitNet: indian fruits image dataset with quality for machine learning applications. Data Br..

[5] Meshram V., Suryawanshi Y., Meshram V., Patil K. (2023). Addressing misclassification in deep learning: a Merged Net approach. Softw. Impacts.

[6] Visvanathan G., Patil K., Suryawanshi Y., Chumchu P. (2023). Sensor based dataset to assess the impact of urban heat island effect mitigation and indoor thermal comfort via terrace gardens. Data Br..

[7] Y. Suryawanshi, V. Meshram, V. Meshram, K. Patil, Face mask wearing image dataset: correct vs. incorrect usage, V2, (2023) doi:10.17632/8pn3hg99t4.2. PMC10700517 38075619

[8] Ottakath N., Elharrouss O., Almaadeed N., Al-Maadeed S., Mohamed A., Khattab T., Abualsaud K. (2022). ViDMASK dataset for face mask detection with social distance measurement. Displays.

[9] Vrigkas M., Kourfalidou E.A., Plissiti M.E., Nikou C. (2022). Facemask: a new image dataset for the automated identification of people wearing masks in the wild. Sensors.

[10] Cabani A., Hammoudi K., Benhabiles H., Melkemi M. (2021). MaskedFace-Net–A dataset of correctly/incorrectly masked face images in the context of COVID-19. Smart Health.

[11] Benini S., Khan K., Leonardi R., Mauro M., Migliorati P. (2019). FASSEG: a FAce semantic SEGmentation repository for face image analysis. Data Br..

[12] Ferioli M., Cisternino C., Leo V., Pisani L., Palange P., Nava S. (2020). Protecting healthcare workers from SARS-CoV-2 infection: practical indications. Eur. Respir. Rev..

[13] Lee L.Y., Lam E.P., Chan C.K., Chan S.Y., Chiu M.K., Chong W.H., Chu K.W., Hon M.S., Kwan L.K., Tsang K.L., Tsoi S.L. (2020). Practice and technique of using face mask amongst adults in the community: a cross-sectional descriptive study. BMC Public Health.

[14] WHO. https://www.who.int/emergencies/diseases/novel-coronavirus-2019/advice-for-public/when-and-how-to-use-masks?adgroupsurvey={adgroupsurvey}&gclid=Cj0KCQjwrMKmBhCJARIsAHuEAPR4b-bNA90_dgiTadWCB8I3fixuHfvLoAGP8hm2sIru3AH5kgsMFKIaAmPrEALw_wcB, 2023 (accessed 07 August 2023).

[15] Centre for Public Protection. https://www.chp.gov.hk/files/pdf/use_mask_properly.pdf, 2023 (accessed 07 August 2023).

